# A novel homozygous mutation in *LSS* gene possibly causes hypotrichosis simplex in two siblings of a Tibetan family from the western Sichuan province of China

**DOI:** 10.3389/fphys.2022.992190

**Published:** 2023-01-06

**Authors:** Bei Zhao, Yisi Tang, Wenjing Chen, Huiying Wan, Jiyun Yang, Xuejun Chen

**Affiliations:** ^1^ Institute of Dermatology and Venereology, Sichuan Academy of Medical Sciences and Sichuan Provincial People’s Hospital, Chengdu, China; ^2^ School of Medicine, University of Electronic Science and Technology of China, Chengdu, China; ^3^ Medical Genetics Center, Sichuan Academy of Medical Sciences and Sichuan Provincial People’s Hospital, Chengdu, China

**Keywords:** hypotrichosis simplex, whole exome sequencing, LSS mutation, autosomal recessive diseases, structure prediction

## Abstract

**Aim:** Hypotrichosis simplex (MIM 146520) is a rare form of monogenic hereditary alopecia. Several genes have been identified as being associated with the disease, including *LPAR6*, *LIPH*, and *DSG4. LSS* encoding lanosterol synthase (LSS) has been shown to cause hypotrichosis simplex, but the related mechanisms have not been elucidated to date. This study aims to find mutations in *LSS* from a Chinese family, among which a 21-year-old male patient and his 9-year-old sister were affected by hypotrichosis simplex.

**Methods:** Dermoscopy and histological analysis were used to examine patients’ scalps, while exome sequencing was used to find the mutations in *LSS*.

**Results:** The hair loss was only detected on the scalp of the proband and his sister, while other ectodermal structures were normal with no systemic abnormalities. Further, the exome sequencing identified a new homozygous mutation NM_002340.6 (LSS_v001):c.812T>C (p.(Ile271Thr)) in the *LSS* gene of the proband, which was also found in his sister. In addition, a heterozygous mutation of *LSS* was found in their asymptomatic parents. Finally, the possible protein structure of the mutational LSS was predicted.

**Conclusion:** The hypotrichosis simplex reported here could be an autosomal recessive disease in this family. The mutation on *LSS* might reduce the enzyme activity of LSS, thus leading to the disease.

## Introduction

Hypotrichosis simplex (MIM 146520) is a rare form of monogenic alopecia. Patients with hypotrichosis simplex usually have normal hair at birth and begin to lose scalp and body hair in early childhood (Romano et al., 2018; Hua et al., 2021). Several genes have been identified as being associated with hypotrichosis simplex, including *LPAR6*, *LIPH*, and *DSG4* (Kljuic et al., 2003; Kazantseva et al., 2006; Pasternack et al., 2008). Normally, lanosterol synthase is a crucial component in the cholesterol synthesis pathway for cholesterol biosynthesis, converting (*S*)-2,3-epoxysqualene to lanosterol. Biallelic mutations in *LSS* were first reported in families with congenital cataracts (Zhao et al., 2015; Romano et al., 2018). More recently, several studies have identified mutations in the lanosterol synthase encoding gene *LSS* from patients affected by hypotrichosis simplex, suggesting *LSS* could also be associated with the disease (Li et al., 2019; Cesarato et al., 2021; Murata et al., 2021). Nevertheless, the pathophysiological mechanism of how its mutations cause hypotrichosis simplex remains unclear.

Mutation analyses in mice and rats confirmed that *LSS* was a causative gene for alopecia and cataracts (Mori et al., 2006; Wada et al., 2020). Similarly, patients with different *LSS* mutations always present different symptoms, such as alopecia, alopecia-mental retardation syndrome, or cataracts (Besnard et al., 2019; Zhao et al., 2021). It was once hypothesized that mutations occurring at the N terminal of LSS would cause hair loss while mutations at the C terminal would lead to cataracts (Romano et al., 2018). The hypothesis has been shown to be flawed with the discovery of novel mutations; thus, new explanations are required to clarify the correlation between genotype and phenotype. In this study, through exome sequencing and Sanger sequencing validation, we identified a new mutation of *LSS* from a Chinese family, among which a 21-year-old male patient and his 9-year-old sister were affected by hypotrichosis simplex. Our work provided new insights into the etiology and pathogenesis of this genetic disease.

## Materials and methods

### Ethics statement and cases collection

Ethical approval for this study was obtained from the Ethics Committee of the Sichuan Academy of Medical Science and Sichuan Provincial People’s Hospital. This study was conducted in compliance with the principles of the Declaration of Helsinki. All the patients provided written informed consent before the study. The proband patient was a 21-year-old male patient in the clinic, who was included in the subsequent studies along with his parents and 9-year-old sister.

### Dermoscopy and histological analysis

Dermoscopy (CH-DS50, Chuanghong Medical Technology Co., Ltd., Guangzhou, China) was used to examine patient scalps. Skin samples from patients were fixed with 4% paraformaldehyde solution and embedded in paraffin, then cut into 5 μm sections. Hematoxylin and eosin (H&E) staining was performed according to the manufacturer’s instructions (Solarbio, Beijing, China).

### Exome sequencing

First, blood samples were collected from the proband. The genomic DNA of the peripheral blood was extracted according to standard procedures followed by sonication, and the fragments were subjected to end-repair and adaptor-ligation. After size selection, the library was constructed and purified by an MGIEasy Exome Capture V4 Probe Set and sequenced on an MGISEQ-2000. Reads were compared with the UCSC hg19 human reference genome by BWA (https://genome.ucsc.edu/). Base quality score, single nucleotide variants (SNVs), and indel were analyzed by GATK (https://gatk.broadinstitute.org/hc/en-us). The variants were prioritized based on the following filtering criteria: 1) The variations with minor allele frequencies (<0.1%) were identified from the following databases: exome aggregation consortium (ExAC), 1000 Genomes Project, and Genome Aggregation Database (Genome AD V3). 2) Synonymous and non-coding variants not predicted to affect splicing were excluded. 3) Variants with an embryogenesis-related function. 4) Location in or near a homozygous region higher than 2.0 Mb. The pathogenicity of each genetic variation was classified based on the Online Mendelian Inheritance in Man (OMIM) (https://www.omim.org/), Human Gene Mutation Database (HGMD) (http://www.hgmd.cf.ac.uk/ac/index.php), ClinVar database (https://www.ncbi.nlm.nih.gov/clinvar/), and the American College of Medical Genetics and Genomics (ACMG) and the Association for Molecular Pathology (AMP) guidelines.

### Sanger sequencing

Blood samples were collected from the proband, his sister, and his parents. DNA was extracted, followed by PCR through high-fidelity DNA polymerase following the protocol (P505-d1, Vazyme Biotech Co., Ltd., Nanjing, China). PCR products were purified by FastPure Gel DNA Extraction Mini Kit (DC301-01, Vazyme Biotech Co., Ltd., Nanjing, China), and Sanger sequencing was performed (Genewiz, Suzhou, China). Primers used for PCR and sequencing were LSS_cds8F: AAT​GAG​CTT​CTG​GGC​AGA​CC and LSS_cds8R: CTC​TGA​GCT​CCT​CCT​TCC​CT.

### Protein structure analysis

UCSF Chimera software was used to visualize 3D models and to predict the consequences of the mutated residues (Couch et al., 2006). The LSS protein model was built from the structure downloaded in the Protein Data Bank as entry 1W6K.

## Results

### A pair of siblings from a Chinese family affected by hypotrichosis simplex

**FIGURE 1:**
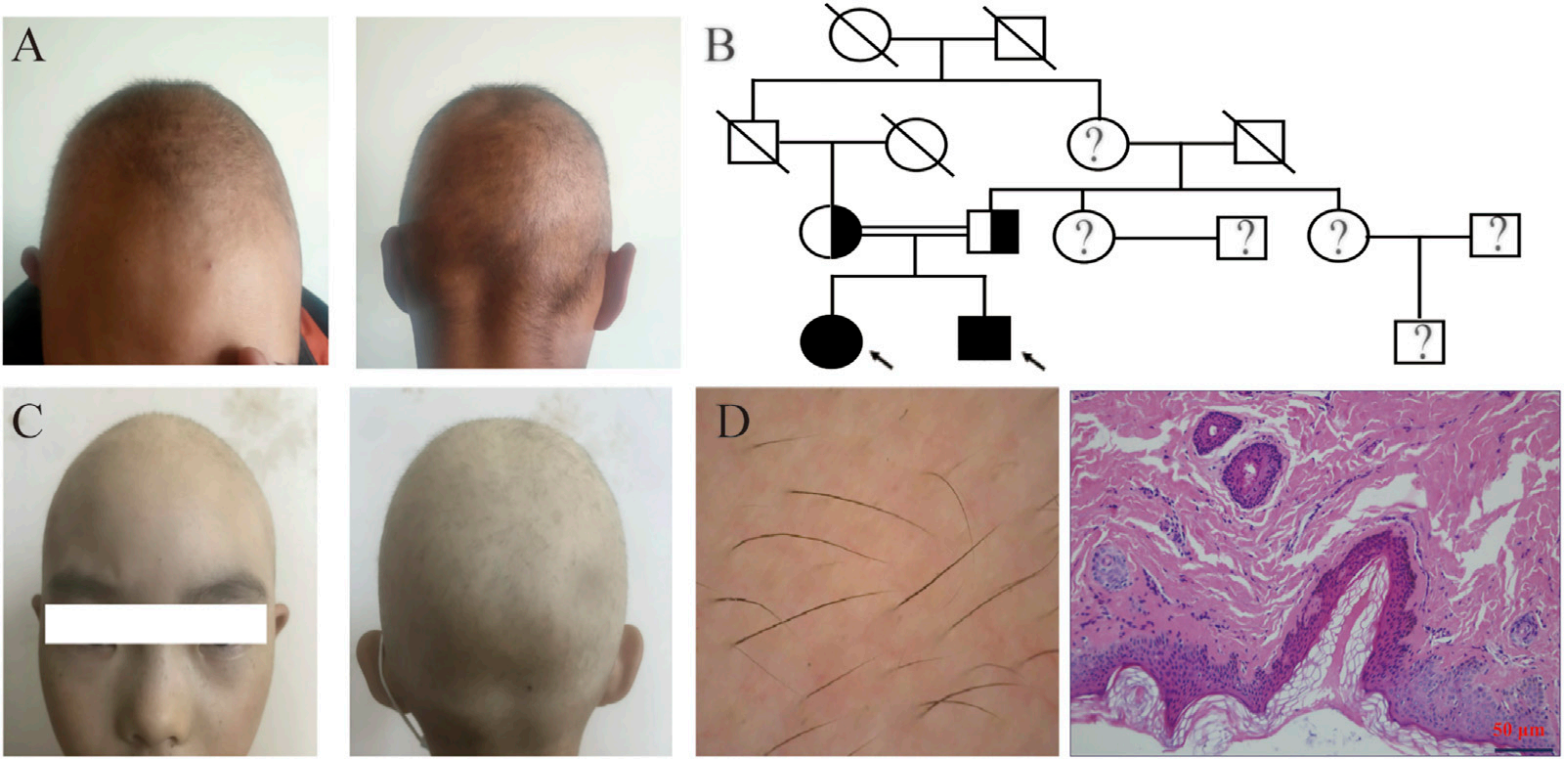
Clinical features of the patients. **(A)** Clinical pictures of the proband showing sparse and thin scalp hair and normal eyebrows. **(B)** Pedigree analysis of the family affected by the *LSS* mutation. The arrow points to the proband; black and white represent mutant and wild type; circles and squares denote females and males; soliduses represent deceased family members; and question marks denote family members not included in this study. **(C)** Clinical pictures of the proband’s sister. **(D)** Skin examination through dermoscopy (left) and skin biopsy (200×) (right).

The proband was a 21-year-old man who presented with sparse scalp hair. He had normal hair at birth but started to lose his hair at 6 months old and has had hair shorter than 0.5 cm since he was 1 year old. Physical examination revealed lightly pigmented hair with slightly reduced strength (Figure 1A). Otherwise, the patient was healthy, including his eyelash, eyebrow, and body hairs. In addition, his nails, intelligence, and development were normal. The proband’s parents were intermarried, and his father informed us that his hair was sparse at birth but became normal after 3 years old (Figure 1B). Meanwhile, the proband has a 9-year-old sister who also experienced sparse hair but had no ocular disorder or intellectual disability (Figure 1C).

### Dermoscopy and histology analysis of the patients

Dermoscopy on the proband’s scalp showed that the patient had diffuse hair thinning without any morphological abnormalities. In addition, most of his hair follicles contained only a single hair, and vellus hairs were few with thin hair shafts and light color. No black dots, broken hairs or exclamation mark hairs could be found. Histological examination showed that epidermal thickness was normal, and the basement membrane was intact despite the decreased number of hair follicles, shown in Figure 1D (Supplementary Material S1). Given that hair loss was only detected on the scalp while other ectodermal structures were normal with no systemic abnormalities, the diagnosis of hypotrichosis simplex was established.

### Genetic screen identifying a new mutation in *LSS*

**FIGURE 2:**
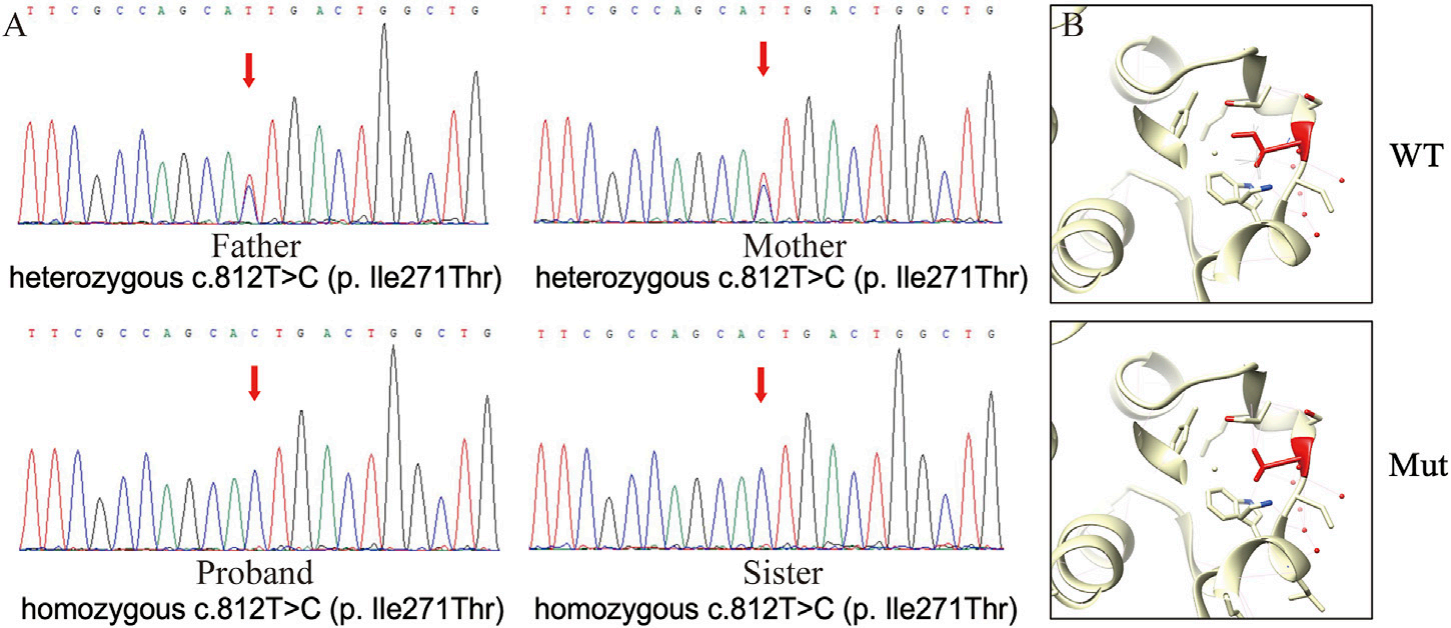
Mutation analyses of the LSS gene. **(A)**. Partial sequences of *LSS* NM_002340.6 (LSS_v001):c.812T>C (p.(Ile271Thr)) mutation. The red arrow indicates the mutation site. **(B)**. 3D modeling of the impact of the LSS nonsense mutation. The red amino acid indicates the mutation site.

A blood sample from the proband was collected for exome sequencing. As a result, a new homozygous mutation locus in the *LSS* gene, NM_002340.6 (LSS_v001):c.812T>C (p.(Ile271Thr)), was identified and confirmed by Sanger sequencing (Figure 2A). Notably, the same homozygous mutation was also found in the proband’s sister, while their parents carried heterozygotes of this mutation (Figure 2A). The allele frequency in GnomAD is 0.00001208 (3 alleles counted among 248,244 alleles), and no other mutation was found associated with alopecia here. According to the Genetic and Rare Diseases Information Center, hypotrichosis simplex is an autosomal dominant inherited disease that can be caused by multiple gene variants, including LSS (https://rarediseases.info.nih.gov/diseases/9170/hypotrichosis-simplex). In addition, different *LSS* mutations have been found in patients affected by hypotrichosis simplex previously (Cesarato et al., 2021; Hua et al., 2021). Therefore, this novel mutation could be pathogenic and related to hypotrichosis simplex.

### The possible relationship between mutational *LSS* and hypotrichosis simplex

Analysis of wild-type and mutated LSS proteins found that Ile271 was located on the surface of the protein and not bound to lanosterol directly (Figure 2B). The hydrophobic Ile271 was near the polar channel of the enzyme surface; mutation into hydrophilic Thr would affect the substrate entering the catalytic center, thus hampering enzyme activity. *LSS* mutations have been identified in patients with cataracts or hypotrichosis. Although its first report was associated with cataracts, most mutation sites were found to cause hypotrichosis (Table 1). Interestingly, for hypotrichosis, mutation sites could be located in either the N-terminal or C-terminal. In this study, protein structure comparison suggested this mutation might reduce LSS enzyme activity, thus possibly leading to hypotrichosis simplex.

**TABLE 1:** Review of LSS mutations.^a^

Disease	Mutation				
Hypotrichosis simplex	P.L102V	P.L248P	P.A399E	P.R568W	p.R234Pfs*2
	P.R177Q	P.I271T	P.R435C	P.W629R	r.425-547del
	P.N209Y	P.G352R	P.V487E	p.W141*	
	P.V236M	P.F391S	P.P549L	p.Y237*	
Hypotrichosis with other symptoms	P.G12D	P.Y286C	P.N516S	p.R604*	
	P.Y14C	P.R294L	P.T652I	p.H473Pfs*32	
	P.R260P	P.R312W	P.T705K		
Cataract	P.I342S	P.W581R	P.G588S	P.W629C	

^a^ The details about the mutations are available at https://cancer.sanger.ac.uk/cosmic/.

*, Nonsense mutation.

## Discussion

Hereditary hypotrichosis simplex is a rare form of alopecia characterized by the onset of hairlessness over the scalp or body from childhood. The phenotype of hypotrichosis simplex is highly heterogeneous as onset time and affected body sites vary greatly among patients. Both autosomal dominant and recessive modes have been reported for hypotrichosis simplex (Romano et al., 2018). For example, *CDSN* was the first reported autosomal dominant hypotrichosis simplex-associated gene whose nonsense mutation was identified in three families who experienced hairlessness over the scalp (Levy-Nissenbaum et al., 2003; Dávalos et al., 2005). In addition, three new genes were identified in patients with autosomal recessive hypotrichosis, including *DSG4* mapped to 18q12,1, *LIPH* to 3q27.3, and *P2RY5* to 13q14.11-13q21.33 (Kljuic et al., 2003; Kazantseva et al., 2006; Pasternack et al., 2008). More hypotrichosis simplex-associated mutations have been found in at least ten genes, including *APCDD1*, *RPL21*, *SNRPE*, *ERCC2*, *KRT25*, *RB1*, and *LSS* (Shimomura et al., 2010; Romano et al., 2018; Ahmed et al., 2019). Despite these breakthroughs, no treatment for hypotrichosis simplex is available due to the phenotypic variations. Here, we identified a novel mutation in *LSS*. LSS is a key enzyme in the cholesterol biosynthesis pathway that catalyzes the first step of the biosynthesis of cholesterol, steroid hormones, and vitamin D. Recently, mutations in *LSS* have been reported to be associated with congenital cataracts, hypotrichosis simplex, and alopecia-mental retardation syndrome (Zhao et al., 2015; Chen and Liu, 2017; Romano et al., 2018; Besnard et al., 2019). Notably, lanosterol, which is the product of *LSS*, could alleviate cataracts by decreasing protein aggregation and increasing transparency in lenses (Zhao et al., 2015). Although epidermal *LSS* knockout led to transient epilation in mice (Wada et al., 2020), the molecular mechanisms of *LSS* mutations related to hair loss remain unclear.

Lipid metabolism is tightly connected to hair growth. The structure and function of hair follicles originating from the skin are regulated by lipid components (Evers et al., 2010). For example, hormones such as testosterone and progesterone are essential for hair growth (Evers et al., 2010). In addition, cholesterol stiffens the cell membrane of the hair follicles and inhibits the diffusion of molecules such as O_2_, CO_2_, and water through the cells (Nguyen and Brownell, 1998). Cholesterol precursors also function as signaling factors and participate in the hair growth cycle. Nevertheless, no obvious decrease in cholesterol and its intermediates has been found in the blood of patients with an *LSS* mutation (Romano et al., 2018; Wada et al., 2020). Consistently, no liver dysfunctions or other lipid metabolism-associated disorders were observed in patients reported here, suggesting that partial rather than systematical lipid metabolism may affect hair loss. This hypothesis is supported by the evidence that inducible epidermis-specific deletion of *L*SS causes alopecia (Fan et al., 2017; Wada et al., 2020). The function of LSS depends on its catalytic activity and accurate cellular location in the endoplasmic reticulum membrane. The three-dimensional structure of LSS suggests the importance of amino acid-forming channels that allow importing and exporting of hydrophobic substrates (Levy-Nissenbaum et al., 2003). Ile271 and Asn209 are in the polar and non-polar regions of LSS, respectively, both of which could reduce enzyme activity without abolishing its total enzyme function. Consistently, we identified an Ile271Thr mutation in the LSS of two patients in this study, suggesting the important role of Ile271 in the enzyme activity of LSS.

Notably, hypotrichosis simplex has an autosomal dominant form and an autosomal recessive form. Different genes contribute to different types of hypotrichosis simplex. Bi-allelic mutations of *LSS* were found to lead to alopecia in all cases reported earlier (Romano et al., 2018; Besnard et al., 2019; Cesarato et al., 2021; Hua et al., 2021). Although we found two patients carried homozygous mutations in this study, their parents were heterozygous with only one mutation in *LSS*. Based on their description, the proband’s parents were intermarried. His father had sparse hair at birth, but it became normal after 3 years. Meanwhile, the proband’s mother was asymptomatic, raising the question of whether a mutation in *LSS* has a dose effect on hypotrichosis simplex, which is worth investigating. It is unclear whether similar situations were observed in other studies, but knockout of *LSS* in mice provided a valuable model that heterozygous mice had no obvious abnormality (Wada et al., 2020). Although we cannot ignore the differences between humans and mice, it is still convincing proof that *LSS*-caused alopecia could be an autosomal recessive disease.

## Conclusion

This study aimed to find new mutations in *LSS* related to hypotrichosis simplex. We examined a Chinese family in which a 21-year-old male patient and his 9-year-old sister were affected by the disease. Through dermoscopy and histological analysis, as well as exome sequencing, we found that hair loss was only detected on the scalp of the patient and his sister while other ectodermal structures were normal, and we identified and validated a new homozygous mutation NM_002340.6 (LSS_v001):c.812T>C (p.(Ile271Thr)) in the *LSS* gene of the patient and their sister. A protein structure comparison suggested this mutation might reduce LSS enzyme activity. In conclusion, the hypotrichosis simplex reported here could be an autosomal recessive disease in this family, and the mutation on *LSS* might reduce LSS enzyme activity, thus leading to alopecia. In the future, the mice model can be utilized to further explore the relationship and mechanisms between the *LSS* mutation and hypotrichosis simplex.

## Data Availability

The original contributions presented in the study are included in the article/Supplementary Material; further inquiries can be directed to the corresponding author.
