# Supplementary figures and images for: A novel homozygous mutation in LSS gene possibly causes hypotrichosis simplex in two siblings of a Tibetan family from the western Sichuan province of China
Source: Front Physiol. 2023 Jan 6;13:992190. doi: 10.3389/fphys.2022.992190 (PMC9859656; doi:10.3389/fphys.2022.992190)

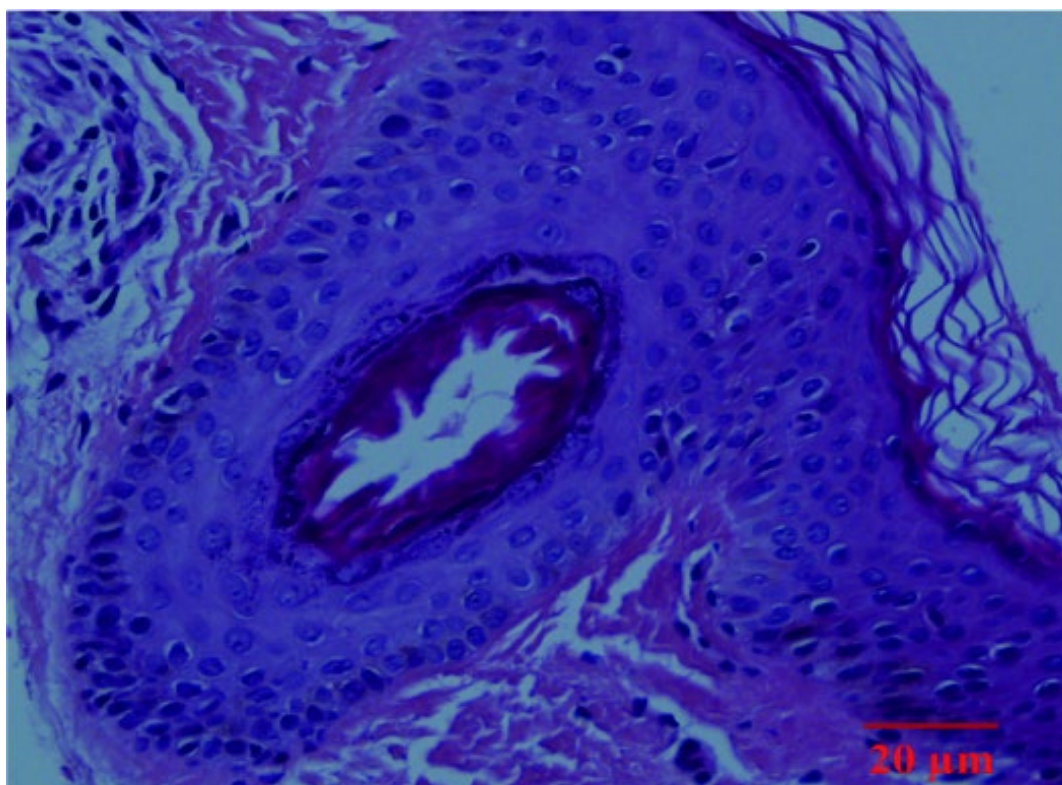

Supplementary Materials S1. Skin biopsy (400X)

Supplement: Supplementary file 1 [file Image1.pdf]
